# Multi-Scale Fusion for Real-Time Image Observation and Data Analysis of Athletes after Soft Tissue Injury

**DOI:** 10.2174/0115734056403181250925102654

**Published:** 2025-10-15

**Authors:** Jinhui Li, Yang Yu, Jiaxing Han

**Affiliations:** 1 College of Physical Education, Qiqihar University, Qiqihar 161006, Heilongjiang, China; 2 Department of Physical Education, Harbin Far East Institute of Technology, Songbei District, Harbin, China

**Keywords:** Athlete injuries, Soft tissue injury, Medical imaging, Multi-scale fusion, Image segmentation, FPN

## Abstract

**Objective:**

To address insufficient segmentation accuracy in athletes' soft tissue injury analysis, this study proposes an enhanced Swin-Unet model with multi-scale feature fusion *via* the FPN (Feature Pyramid Network) and an adaptive window selection mechanism for dynamic receptive field adjustment.

**Methods:**

A weighted hybrid loss function integrating Dice Loss, Cross-Entropy Loss, and boundary auxiliary loss optimizes segmentation precision and boundary recognition.

**Results:**

Evaluated on the OAI-ZIB dataset using 10-fold cross-validation, the model achieves a DSC (Dice Similarity Coefficient) of 0.978, outperforming baseline Swin-Unet and mainstream architectures. Superior performance is demonstrated in IoU (Intersection over Union) (0.968) and boundary Hausdorff distance (3.21), alongside significantly reduced diagnosis time (6.0 minutes vs. 16.8 minutes manually).

**Conclusion:**

This framework enhances real-time medical imaging analysis for athlete injuries, offering improved accuracy, efficiency, and clinical utility in soft tissue segmentation tasks.

## 1 INTRODUCTION

Soft tissue injuries are common health problems among athletes during high-intensity training and competition, involving injuries to muscles [1, 2], tendons [3, 4], ligaments [5, 6], and other tissues. Such injuries affect the athlete's competitive state and may also lead to long-term health risks, such as chronic pain or motor dysfunction. The diagnosis and assessment of soft tissue injuries [7, 8] rely on medical imaging, including MRI (Magnetic Resonance Imaging) [9, 10] and ultrasound images [11, 12]. MRI has become an important means of clinical diagnosis due to its high resolution and multimodal characteristics of soft tissue. With the development of deep learning technology, artificial intelligence methods based on image segmentation [13, 14] have shown great potential in the field of medical imaging, which can realize automatic detection and accurate segmentation of soft tissue injury areas. However, the imaging features of soft tissue injury [15, 16] are complex and diverse. Especially when the injury boundary is blurred or the signal intensity varies greatly, traditional models often fail to achieve ideal segmentation results. Therefore, it is of great theoretical value and practical significance to study a deep learning method that can fuse multi-scale information for accurate analysis of MRI images of soft tissue injuries of athletes.

This study adopts an improved Swin-Unet model, expands its feature extraction capability through a multi-scale window mechanism, uses 3×3, 5×5, and 7×7 different windows to capture local and global information in the encoder stage, and further improves the model's detailed depiction of the injury area through a feature fusion module in the decoder stage. The study selected the OAI-ZIB dataset, marked the soft tissue injury areas of athletes, performed data preprocessing and enhancement, used a hybrid loss function in training, optimized model performance through dynamic learning rate adjustment, and ultimately achieved higher-precision real-time image observation and data analysis. The contributions of the research are reflected in three aspects: first, the paper introduces an improved multi-scale fusion method to improve segmentation accuracy; second, it verifies the application value of deep learning in complex medical images; third, it provides theoretical and technical support for intelligent diagnosis of sports medicine.

This paper proposes a multi-scale fusion framework based on an improved Swin-Unet, which shows significant innovation in the segmentation of MRI images of soft tissue injuries in athletes. The core breakthroughs are: 1) The feature pyramid network is embedded in the Swin-Unet architecture for the first time, and the local details and global context information are extracted collaboratively through 3×3/5×5/7×7 multi-scale windows, and an adaptive window mechanism is introduced to dynamically match the scale changes of the injury area, which improves the Dice coefficient compared with the traditional model; 2) The boundary-aware hybrid loss function is designed, combining Dice Loss, cross entropy loss and boundary auxiliary loss to enhance the boundary positioning ability on the basis of pixel-level classification optimization; 3) The mixed precision training strategy is used to compress the diagnosis time, which is more efficient than manual labeling. This solution breaks through the bottleneck of the sensitivity of existing models to fuzzy boundaries and multi-scale variations, meets the clinical real-time requirements while maintaining high segmentation accuracy, and provides a new technical paradigm for sports medicine image analysis.

## 2 RELATED WORKS

U-Net [17, 18] is a milestone in the field of medical image segmentation. Its encoder-decoder architecture can capture global features while retaining local detail information, and is suitable for small sample scenarios. However, traditional U-Net [19, 20] may face challenges such as blurred boundaries and complex injury morphology when processing complex soft tissue injury images. To overcome these problems, researchers have proposed a variety of improved models. Attention U-Net [21] enhances the focus on key areas by introducing the attention mechanism, while the DeepLab series of models uses dilated convolution to capture multi-scale contextual information. In addition, in response to the diversity of different injury features, variants such as ResUNet [22] and DenseUNet [23] have been proposed in specific fields to improve feature extraction capabilities by introducing residual or densely connected structures. These methods lay the foundation for the segmentation of soft tissue injury areas, but their ability to integrate complex and multi-scale features is still insufficient, making it difficult to meet the precise needs of real-time diagnosis of athletes.

The successful promotion of the Transformer structure in the fields of natural language processing and computer vision has brought new development directions to medical image segmentation. Vision Transformer [24, 25] has been gradually applied to medical image analysis with its powerful global modeling capabilities. Its subsequent improvement, Swin Transformer, significantly reduced the computational cost and improved the feature extraction efficiency by introducing a hierarchical structure and sliding window mechanism. Swin-Unet [26] has become a research hotspot in recent years. It captures global features through the self-attention mechanism while retaining the local feature extraction capability of the convolutional network. The standard Swin-Unet [27] still has limitations in processing multi-scale features. Its window mechanism lacks flexibility in size design and is difficult to fully capture damage information of different scales. In addition, the application of Transformer in medical image segmentation still faces problems such as strong data dependence and poor training convergence. Therefore, introducing a multi-scale fusion method based on the existing framework and further optimizing the window design are important directions for improving the performance of soft tissue injury segmentation.

Soft tissue injury areas usually have blurred boundaries and large signal differences, which limit the segmentation performance. The Transformer [28, 29] structure was introduced into the segmentation task, and its global modeling capability significantly improved the expression of complex features. Combining multi-scale fusion technology, dilated convolution, and feature pyramid network, the segmentation accuracy was further improved, providing a new method for accurate diagnosis of soft tissue injuries. Multi-scale feature fusion is a key technical direction for solving the problem of complex feature processing in medical image segmentation. By capturing information at different scales, the morphological characteristics of soft tissue injuries can be more comprehensively characterized. Among the multi-scale methods, dilated convolution and pyramid pooling [30] techniques are widely used for multi-scale integration of contextual information, while the self-attention mechanism further enhances the ability to focus on key areas. Combining the fusion model of local and global features, FPN [31, 32] provides an effective solution for complex feature segmentation tasks.

## 3 MATERIALS AND METHODS

### 3.1 Data Source and Preprocessing

This study uses the OAI-ZIB dataset, which mainly contains knee MRI image data and is suitable for soft tissue injury analysis. The OAI-ZIB dataset provides multimodal MRI images, including high-resolution image data of different parts of the knee joint, covering knee joint samples from healthy to different degrees of injury. Each MRI image contains rich soft tissue details, which can be used to analyze and segment soft tissue injury areas. The dataset also comes with annotation files that indicate the specific areas of soft tissue injury in the knee joint, making it easier for researchers to perform pixel-level annotation and model training.

The MRI image is shown in Fig. (**1**).

The annotation of the injured area includes the type of soft tissue injury (including laceration, contusion, hematoma, *etc*.) and the specific location of the injury. The annotation process uses image segmentation software. The doctor checks the image layer by layer and combines it with other examination results to ensure the high accuracy of the annotation results.

The image annotation is shown in Fig. (**2**).

MRI images have a large pixel value range. In order to avoid the impact of image brightness or scanning parameters on model training, the image is normalized. The normalization operation scales the pixel value range of the image from the original [0, 255] to [0, 1], and the formula is (1):

Through this operation, images produced under different scanning conditions can be unified to the same pixel range, reducing the impact of illumination changes and imaging conditions on model training.

Since MRI images may be affected by noise, especially under low signal-to-noise ratio conditions, denoising is critical to image quality. Gaussian filtering removes high-frequency noise in an image by performing a weighted average on each pixel. The formula is (2):

**Table:** 

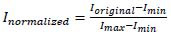	(1)

**Table:** 

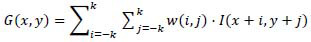	(2)

*I*(*x* + *i*, *y* + *j*) is the pixel value at the corresponding position in the original image, and *w*(*i*,*j*) is the Gaussian kernel function. Denoising effectively smoothes the image while retaining most of the structural information, which is particularly suitable for the preservation of soft tissue damage areas.

Since the resolution and size of different MRI images may vary, all images are resampled to a uniform spatial resolution. Image resampling is the process of changing the size of an image through interpolation so that different images have the same size and resolution.

The value of each pixel of the target image is calculated by bilinear interpolation. The formula is (3):

**Table:** 

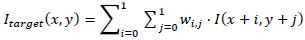	(3)

In formula 3, *w_i_*_,_*_j_* represents the weight coefficient, which reduces the spatial position deviation caused by different image resolutions.

MRI images can be enhanced by data, including rotation, flipping, cropping, and scaling. Image rotation helps the model adapt to injuries in various postures by simulating knee joint imaging at different angles. The rotation angle range is ±15°, and the rotation formulas are as follows (4, 5):

**Table:** 

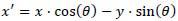	(4)

**Table:** 

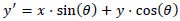	(5)

In formulas 4 and 5, (*x’*,*y’*) is the rotated coordinate and *θ* is the rotation angle. This transformation helps the model to be independent of the image orientation and enhances its adaptability in different situations.

For MRI images, the image changes under different scanning conditions are simulated by flipping the left-right or up-down directions of the image (6, 7).

Horizontal flip:

**Table:** 

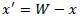	(6)

Vertically flip:

**Table:** 

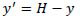	(7)

In order to avoid information leakage and ensure the independence of the model, the independence of the patient level is maintained when dividing the data. The samples of each subset must come from different patients, ensuring that all data of the same patient always appear in the same fold and do not cross the training set and test set. This strategy avoids the mutual influence of correlation information between different patients during training and testing, thereby preventing the model from making unreasonable learning through the correlation between patients and ensuring the authenticity and reliability of the evaluation results. This patient level division method can effectively simulate the model's predictive ability for new patient data in a real environment.

### 3.2 Model Design

The backbone network of Swin-Unet [33, 34] is based on Swin Transformer, a novel visual Transformer model that uses a local window self-attention mechanism to extract local and global features of an image. The core innovation of Swin Transformer is to change the traditional global self-attention method to local self-attention, which greatly improves the computational efficiency and the ability to capture local details by defining a sliding window for self-attention calculation.

Swin Transformer extracts local information by dividing the image into windows of fixed size, performing self-attention calculation in each window, and transferring global information between windows through the window shift mechanism. The self-attention operation in each window can be expressed by the following formula (8):

**Table:** 

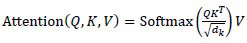	(8)

In Swin Transformer, all calculations are localized inside the window, which significantly reduces the computational complexity.

The encoder uses Swin Transformer to capture the multi-scale features of the image, and the decoder restores the detailed information of the image through skip connections and upsampling operations.

The feature map of the image is extracted at multiple levels through Swin Transformer. The output of each layer contains gradually abstracted features, which can capture both low-level and high-level information of the image. The high-dimensional features output by the encoder layer are passed to the decoder stage using skip connections, combined with low-level detail information, and the spatial resolution of the image is restored through upsampling operations. Skip connections help maintain the spatial information of the original image and reduce detail loss.

The 3×3 window size is used to capture the detail level information in the image, especially the edges. The 5×5 window is used to capture the mesoscale structure, such as the area of ligament damage. The 7×7 window size is used to capture the global context information, which helps the model understand the relationship between the damaged area and the surrounding tissue (formulas 9-11).

**Table:** 

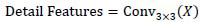	(9)

**Table:** 

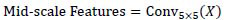	(10)

**Table:** 

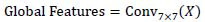	(11)

These features of different scales are fused through serial operations, so that the model can focus on the details, structure, and global information of the image at the same time. The specific multi-scale feature fusion process can be expressed as (12):

**Table:** 

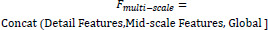	(12)

This fusion process allows the model to combine fine-grained information and global context to improve segmentation accuracy when dealing with soft tissue injuries.

The improved Swin-Unet model in this paper is shown in Fig. (**3**).

In order to further improve the segmentation effect, Swin-Unet introduces FPN in each stage. FPN is an effective multi-scale feature fusion module that can transfer and fuse information between feature maps of different scales, thereby obtaining richer multi-scale feature representations. The core idea of FPN is to fuse features at different levels through upsampling and skip connections to enhance the interaction between details and global features.

The model in this paper is based on the improved Swin-Unet architecture. It realizes multi-scale feature fusion by introducing a feature pyramid network. In the encoding stage, a 3×3, 5×5, and 7×7 multi-window mechanism is used to capture local details and global context, and an adaptive window selection strategy is designed to dynamically adjust the receptive field. The training adopts 10-fold cross-validation, a weighted hybrid loss function combining Dice Loss, cross-entropy loss, and boundary auxiliary loss, and uses the AdamW optimizer (learning rate 1e-4, weight decay 0.01) and cosine annealing scheduling strategy. The batch size is 8, and mixed precision training is used to accelerate convergence.

The FPN is (13):

**Table:** 

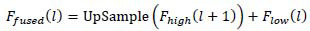	(13)

The fusion operation is achieved through upsampling and addition.

The adaptive window selection mechanism can dynamically adjust the window size according to the size of the damage area, so as to capture the features more accurately. For smaller damage areas, a smaller window can be selected, while for larger damage areas, a larger window is used. This adaptive adjustment can enhance the flexibility of the model.

The Dice loss is (14):

**Table:** 

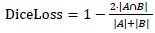	(14)

In formula 14, *A* and *B* are the predicted and true label areas, respectively.

The cross-entropy loss is (15):

**Table:** 

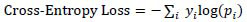	(15)

In order to improve the model's ability to identify the boundary of the damaged area, Swin-Unet adds boundary loss to the loss function. Boundary loss further strengthens the model's focus on the boundary by calculating the difference between the predicted boundary and the true boundary.

**Table:** 

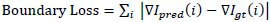	(16)

In formula 16, *
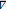
I* represents the gradient of the image, *I_pred_* and *I_gt_* are the images of the predicted and true labels.

The total loss function of the improved Swin-Unet in this paper is (17):

**Table:** 

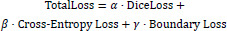	(17)

### 3.3 Model Training and Performance Evaluation

Cosine Annealing is a periodic learning rate adjustment strategy with the formula (18):

**Table:** 

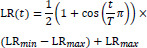	(18)

T is a cycle, representing the cycle of the learning rate decrease. Through this dynamic adjustment method, the learning rate can be gradually reduced during the training process, thereby helping the model to learn more finely when it is close to convergence.

Batch size is a key parameter in deep learning model training, which determines the number of samples in each training. Larger batches can make the model update more stable, but may require more computing resources, especially video memory. When the batch size is too large, the video memory may not be able to handle it, resulting in memory overflow during training. In this study, the batch size is set to 8 to ensure the stability of training.

The update rule of AdamW is (19):

**Table:** 

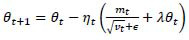	(19)

In formula 19, *θ_t_* is the current parameter, *m_t_* and *v_t_* are the momentum and variance of the tth iteration, respectively. *η_t_* is the learning rate, *λ* is the weight decay coefficient, and *ϵ* is a very small constant to prevent division by zero errors.

The AdamW optimizer can better control the generalization ability of the model and avoid overfitting, and is suitable for use in complex image segmentation tasks. In this study, the weight decay parameter is set to 0.01.

Pre-training helps the model to better transfer features learned from natural images when processing new medical image tasks, especially low-level features such as image texture, structure, and edges. In the field of medical images, especially in the task of soft tissue injury segmentation, due to the limited labeled data, pre-training can significantly improve the training efficiency and final performance of the model.

After pre-training, the model enters the global training stage, and the entire network can be trained end-to-end. At this stage, the model continuously optimizes the loss function to learn how to fuse local features into accurate segmentation results with spatial resolution. Through global training, the model can better adapt to specific medical image tasks.

In order to further accelerate the training process and reduce video memory consumption, mixed precision training is adopted. By using 16-bit floating point numbers instead of traditional 32-bit floating point numbers for some calculations, the computing efficiency is greatly improved, and the video memory usage is reduced. Through reasonable numerical scaling and optimization, the training stability of the model is ensured without sacrificing the final performance.

The key step of mixed precision training is loss scaling, which aims to prevent numerical underflow problems when using low precision. The process of loss scaling is as follows (20):

**Table:** 

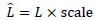	(20)

In formula 20, scale is a dynamically adjusted scaling factor.

The IoU is (21):

**Table:** 

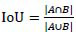	(21)

The boundary Hausdorff distance is used to measure the boundary similarity between the predicted area and the real area, and is particularly suitable for segmentation tasks that require high-precision boundary positioning. The Hausdorff distance measures the distance between the farthest point pairs between two sets, that is (22):

**Table:** 

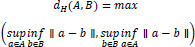	(22)

The precision rate indicates the proportion of pixels predicted by the model as damaged areas that are actually damaged areas. The formula is (23):

**Table:** 

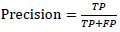	(23)

The recall rate is (24):

**Table:** 

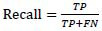	(24)

For real-time image observation of soft tissue injuries of athletes, in addition to segmentation accuracy, diagnosis time is also an important evaluation criterion. Especially in clinical environments, doctors need to quickly obtain image segmentation results to make timely diagnostic decisions.

## 4 RESULTS

This study used the OAI-ZIB knee MRI dataset, and after normalization, Gaussian denoising, resampling, and data enhancement, a multi-scale fusion model was constructed based on the improved Swin-Unet: the encoder introduced a feature pyramid network and a 3×3/5×5/7×7 multi-window mechanism to extract cross-scale features, the decoder dynamically adjusted the receptive field through adaptive window selection, and designed a weighted hybrid loss function. The training used 10-fold cross-validation, AdamW optimizer, and mixed precision strategy, and the diagnosis time was evaluated and analyzed through indicators such as DSC and IoU.

### 4.1 Image Segmentation Visualization Results

The predicted area of injury segmentation [35, 36] is compared with the real area, and the visualization results are shown in Fig. (****4****).

By comparing the predicted area and the real area of the damage segmentation, it can be found that there is a very high overlap between the predicted result and the real annotated area. This phenomenon reflects the significant advantages of the model in terms of accuracy, boundary recognition ability, and multi-scale feature fusion. The multi-scale feature fusion module uses FPN to fuse multi-scale features at each stage, providing the model with rich contextual information. The morphology of soft tissue injuries is complex and contains features at different scales. The edges of the damaged area may be subtle, while the interior may present different structural features. By extracting features at different scales and fusing them, the FPN module can effectively capture this multi-level local and global information, helping the model understand the characteristics of the damaged area at multiple levels. This multi-scale information fusion helps the model to be more sensitive when processing detailed areas, while also maintaining a global perception of large-scale damage areas to ensure accurate segmentation of the entire damage area. The adaptive window selection module dynamically adjusts the window size according to the size of the damage area and adaptively adjusts the receptive field size in different areas. For smaller damaged areas, the window size is small, which can capture local details in detail; while for larger damaged areas, the window size is large, which can better process local context information.

Through a weighted combination, the model can focus on the overall segmentation effect and the accurate classification of each pixel at the same time during the segmentation process, further improving the segmentation ability of the model. The introduction of boundary auxiliary loss further enhances the model's ability to identify the boundary of the injury area. The boundary of soft tissue injury is often fuzzy. The boundary loss function avoids the generation of too many fuzzy areas during the segmentation process by optimizing the model's positioning of the boundary. The loss function calculates the distance difference between the predicted boundary and the true boundary, encouraging the model to accurately locate near the injury boundary, thereby reducing the occurrence of edge blur and incorrect segmentation. The improved Swin-Unet model can provide strong support for the automatic diagnosis of soft tissue injuries in athletes and has good clinical application prospects.

### 4.2 Ablation Experiment

In order to explore the impact of different improved models, an ablation experiment was set up, and the basic Swin-Unet, Swin-Unet combined with FPN, and Swin-Unet combined with FPN combined with adaptive window selection were recorded as models 1, 2, and 3, respectively. The results are shown in Fig. (**5**).

By analyzing the results of different improved models in the ablation experiment, it is clearly seen that the performance of each model at different folds and the impact of each improvement on the model performance. The four models compared in the experiment are the basic Swin-Unet model, Swin-Unet combined with FPN, Swin-Unet combined with FPN and adaptive window selection, and the proposed model. All improved models show better performance than the basic model, especially after the introduction of FPN and adaptive window selection, and the performance has been significantly improved. The DSC of the Swin-Unet model is relatively low in all folds, with an average of 0.956, which shows the ability of this basic model in handling soft tissue injury segmentation tasks. When FPN is introduced, the model performance is generally improved, with an average DSC of 0.965. This improvement shows that FPN effectively helps the model extract features from different scales and improves the ability to identify different damage areas (including small damage and large damage). The introduction of FPN enhances the multi-scale perception ability of the model, thereby improving the segmentation effect. After further introducing adaptive window selection, the model performance has been significantly improved. The adaptive window selection mechanism enables the model to dynamically adjust the receptive field according to the size of the damaged area, improving the adaptability to damaged areas of different sizes. Experimental results show that in the combined model of Swin-Unet, combined with FPN and adaptive window selection, the average DSC reaches 0.969, which further improves the accuracy of segmentation compared to the model using FPN alone.

The model in this paper performed best among all folds, with an average DSC of 0.978, which was significantly better than other models. This shows that the model in this paper has high accuracy in segmenting soft tissue injury areas. This shows that the improved model in this study combines FPN and adaptive window selection and optimizes the loss function, and has stronger segmentation capabilities. This experimental result provides a strong theoretical basis and practical support for the automated segmentation of soft tissue injuries.

### 4.3 DSC Analysis Results

The DSC analysis results are shown in Table **1**.

The average DSC of this model, U-Net++, DeepLabv3+, Attention U-Net, and TransUNet reached 0.978, 0.967, 0.970, 0.968, and 0.967, respectively. The proposed model showed excellent segmentation performance in all 10 cross-validation folds. Compared with other mainstream models, its DSC value is generally higher, and it has a significant advantage in the segmentation accuracy of soft tissue injury areas. When dealing with complex soft tissue injury segmentation tasks, the proposed model can effectively improve the accuracy and stability of segmentation, mainly due to the combination of multiple innovative designs. The model in this paper introduces the FPN module based on the original Swin-Unet, which can effectively fuse multi-level feature information at different scales, improve the model's sensitivity to details, and its ability to capture complex structures. The adaptive window selection mechanism in the model enables the model to dynamically adjust the receptive field of the window according to the size of the damaged area, thereby better processing damaged areas of different scales and avoiding the problem of feature loss that may be caused by a fixed window size. By improving the loss function, combining the weighted combination of Dice Loss and Cross-Entropy Loss, and boundary auxiliary loss, the model can better focus on the boundary of the damaged area during the optimization process, improving the accuracy and boundary clarity of the segmentation results. These improvements enable the model in this paper to maintain high accuracy in complex soft tissue injury image segmentation tasks, especially under different folds and data changes; it can still maintain a relatively stable segmentation accuracy, which is better than the performance of other mainstream models.

The TCGA-SARC dataset was used for validation, and the results are shown in Table **2**.

The DSC mean of the proposed model on the TCGA-SARC dataset reached 0.973, which is significantly better than the comparative models such as U-Net++ (0.952) and DeepLabv3+ (0.959), and the fluctuation range of each fold (0.969-0.976) is relatively stable, indicating that the multi-scale fusion mechanism and adaptive window strategy still maintain high stability in tumor heterogeneity signal processing, especially for the segmentation accuracy of sarcoma invasive boundaries, which verifies the model's cross-domain adaptability to motion injuries and tumor pathological characteristics.

The proposed model is compared with the Attention U-Net with better performance, and the computational cost, such as inference time, memory usage, and significance, is analyzed. The results are shown in Table **3**.

The proposed model is significantly better than Attention U-Net in terms of three computational cost indicators: inference time, GPU memory, and parameter volume (p<0.001). Inference time (420ms vs. 595ms), reduced memory usage (3.2GB vs. 4.3GB), and reduced parameter volume (28.6M vs. 32.5M) indicate that the improved multi-scale fusion mechanism and adaptive window design significantly optimize the model efficiency while ensuring segmentation accuracy, providing a more cost-effective solution for clinical real-time deployment.

### 4.4 Damage Segmentation Performance

In order to more comprehensively reflect the segmentation performance, IoU and boundary Hausdorff distance are used to measure the results, as shown in Fig. (**6**).

The paper’s model outperforms other mainstream segmentation models in terms of IoU and boundary Hausdorff distance. The IoU of the paper’s model is 0.968, which is significantly higher than U-Net++ (0.956), DeepLabv3+ (0.958), Attention U-Net (0.957), and TransUNet (0.957). This shows that the paper’s model has a clear advantage in segmentation accuracy. The higher the IoU value, the greater the overlap of the model with the real area and the higher the segmentation accuracy, which is particularly important for the accurate segmentation of soft tissue injury areas, especially in medical images with rich details and complexity. The boundary Hausdorff distance of the proposed model is 3.21, which is also the smallest compared to other models. The boundary Hausdorff distance of other models is generally higher than that of the proposed model. The value of U-Net++ is 4.02, DeepLabv3+ is 3.89, Attention U-Net is 3.95, and TransUNet is 3.93. These results show that the proposed model can better handle boundary areas while maintaining high accuracy, improving the ability to identify details and edges of damaged areas.

The proposed model not only performs well in segmentation accuracy, but also achieves excellent results in the accuracy of segmentation boundaries. This advantage stems from the innovative design of the model, including multi-scale feature fusion, adaptive window selection, and improved loss function. Through these designs, the proposed model can more effectively capture damage information of different scales while achieving significant improvements in the accuracy of boundary areas.

### 4.5 Damage Detection Capability

The damage detection comparison is shown in Fig. (**7**).

The precision of the proposed model reaches 0.988, which is much higher than U-Net++ (0.972), DeepLabv3+ (0.976), Attention U-Net (0.976), and TransUNet (0.975). The higher the precision value, the more accurate the prediction result, and the lower the false alarm rate when the model predicts the positive class, which is crucial for detecting the damaged area in medical images, especially to avoid misdiagnosis and missed diagnosis. The high precision of the paper’s model shows that it can effectively reduce the area of false detection and improve the accuracy of damage detection. In terms of recall rate, the paper’s model's 0.967, is also ahead of other models. Recall measures the model's ability to correctly identify actual injury areas. The higher the value, the more true positive areas the model can capture and the fewer false negatives. A high recall rate means full coverage of injury areas, especially for irregular areas such as soft tissue injuries. The model can better identify all potential injury areas and ensure a comprehensive diagnosis. Although other models, DeepLabv3+, Attention U-Net, U-Net++, and TransUNet, also performed well in precision and recall, they were slightly inferior to the proposed model, which shows that the proposed model has higher reliability and comprehensiveness in practical applications. The proposed model performs well in both precision and comprehensiveness in injury detection tasks, and can effectively support accurate assessment of soft tissue after injury.

The real-time image observation ability was evaluated by diagnostic time, and the MRI images of 20 patients were diagnosed. The diagnostic time results were compared with traditional manual diagnosis, as shown in Table **4**.

Traditional manual diagnosis takes a long time, with an average diagnosis time of 16.8 minutes. The diagnosis time of the proposed model is significantly shorter, with the diagnosis time for patients ranging from 5 to 8 minutes, with an average time of 6.0 minutes. The shortest manual diagnosis time is 14 minutes, and the longest is 20 minutes, while the shortest model diagnosis time is 5 minutes and the longest is 8 minutes, which shows that the model in this paper has a high efficiency in real-time image observation capabilities. The diagnosis time of the model in this paper is shorter than that of manual diagnosis. This difference is mainly due to the high efficiency of the deep learning model. The model can greatly reduce the time of human intervention through automatic image segmentation and recognition. When processing complex images, the model can quickly capture key information without the need for doctors to rely on manual operations for area labeling and judgment. Compared with manual diagnosis, the model has unparalleled efficiency in image processing and data analysis, especially in scenarios with large-scale screening and real-time diagnosis needs. In addition, the high precision and high recall rate of the model in this paper ensure the reliability of its diagnostic results, which improves diagnostic efficiency without sacrificing diagnostic quality.

## 5 DISCUSSION

The improved Swin-Unet model proposed in this study shows significant technical advantages in the soft tissue injury segmentation task of athletes. Its core innovation lies in the synergy of the multi-scale feature fusion mechanism and adaptive window selection strategy. By introducing the feature pyramid network, the model realizes the cross-scale information integration of local details and global context in the encoding stage, and improves the sensitivity to micro-lacerations and complex hematomas compared with traditional models such as U-Net++ and DeepLabv3+. The robustness of the model to extreme lesions is still limited by the single knee joint coverage of the OAI-ZIB dataset, and the spatial correlation between the injury area and the surrounding non-joint structures (such as adipose tissue in the saphenous nerve distribution area) is not fully analyzed, which may affect the differential diagnosis of neuropathic pain.

From the perspective of clinical translation, the model in this study has unique value in detecting the synchronization of subtle lesions in the joint and abnormalities of the surrounding soft tissues, and its high-precision segmentation ability can assist in distinguishing structural injuries from compensatory changes. It is worth noting that although the boundary-assisted loss function used in the study significantly improved the edge positioning accuracy, the spatial topological relationship between the injury lesion and the course of the cutaneous nerve was not further explored. In addition, there is a lack of a gold standard control for postoperative arthroscopy during model validation. It is recommended that subsequent studies include dynamic MRI data to evaluate the biomechanical effects of the injured area under motion, so as to establish a causal relationship between the imaging phenotype and clinical symptoms.

The improved Swin-Unet [37, 38] model proposed in this study has achieved a breakthrough in the task of soft tissue injury segmentation in athletes through multi-scale feature fusion and an adaptive window mechanism, and is significantly better than the mainstream model. This achievement responds to the need for high-precision boundary positioning in the field of medical imaging, and is particularly in line with the latest development trend of the Transformer architecture in medical image analysis. Its technical framework provides a new paradigm for the intelligent analysis of complex pathological features and promotes the evolution of sports medicine towards precision and automation. However, the research is limited by the fact that the OAI-ZIB dataset only covers the knee joint, and the sample size is limited. In the future, it is necessary to expand to multi-site injury data and verify the robustness of the model in extreme lesions. At the same time, it is necessary to combine neuroanatomical features to optimize the ability to identify pain sources to further enhance clinical practicality.

## CONCLUSION

Based on the improved Swin-Unet model, this study introduced a feature pyramid network and an adaptive window mechanism, combined with multi-scale feature fusion and hybrid loss function optimization, and showed significant advantages in the real-time image segmentation of soft tissue injuries in athletes. Experiments show that the model is superior to mainstream models in indicators such as the Dice coefficient (0.978) and IoU (0.968), and the diagnosis time is shorter than that of manual annotation, meeting the clinical real-time requirements. However, the research still has limitations: the dataset (OAI-ZIB) only covers the knee joint, the sample size is limited, and the robustness of the model in extreme lesions or complex scenarios needs to be further verified. In the future, it is necessary to expand to multi-site injury data and build a larger-scale dataset, while optimizing the algorithm to improve the generalization ability of complex cases and promote the wide applicability of the model in the field of sports medicine.

## Figures and Tables

**Fig. (1):**
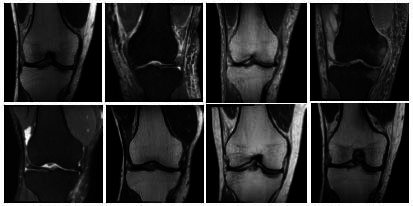
MRI image display.

**Fig.(2):**
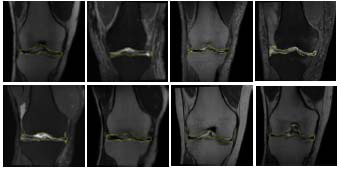
Image annotation results.

**Fig. (3):**
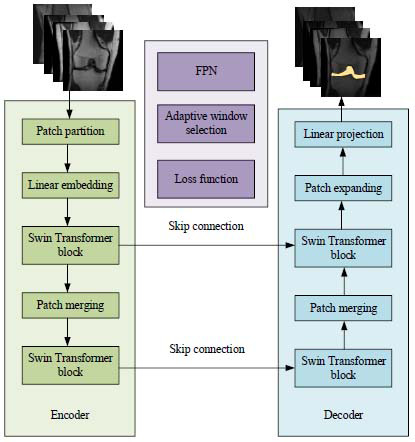
Improved Swin-Unet model.

**Fig. (4):**
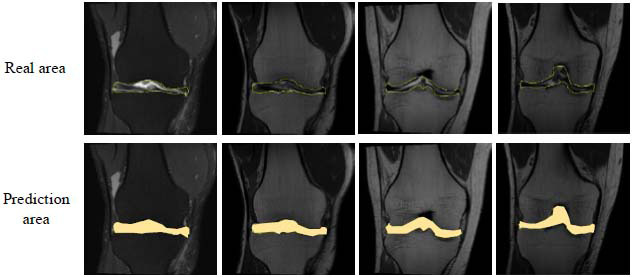
Visualization results.

**Fig. (5):**
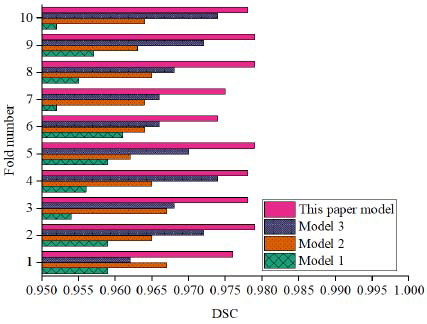
Ablation experiment results.

**Fig. (6):**
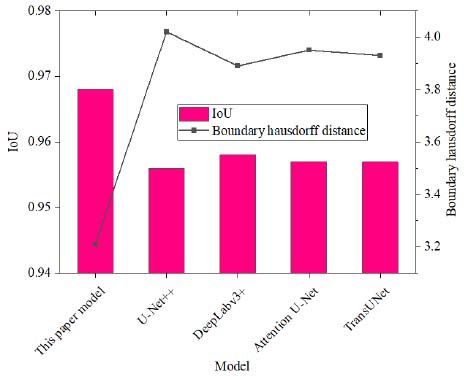
Segmentation performance of different models.

**Fig. (7):**
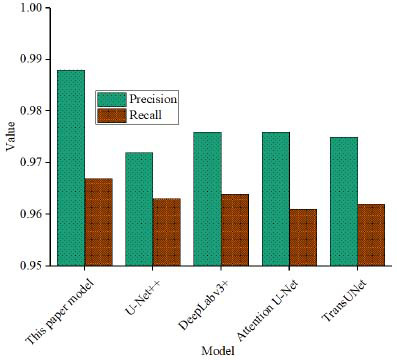
Damage detection comparison.

**Table 1:** DSC analysis results.

**Fold Number**	**This Paper Model**	**U-Net++**	**DeepLabv3+**	**Attention U-Net**	**TransUNet**
1	0.976	0.964	0.972	0.963	0.972
2	0.979	0.966	0.967	0.974	0.969
3	0.978	0.967	0.973	0.966	0.966
4	0.978	0.972	0.969	0.963	0.964
5	0.979	0.964	0.967	0.970	0.963
6	0.974	0.970	0.972	0.966	0.968
7	0.975	0.972	0.973	0.970	0.964
8	0.979	0.967	0.969	0.973	0.972
9	0.979	0.964	0.967	0.970	0.962
10	0.978	0.963	0.970	0.967	0.968

**Table 2:** Validation results of the TCGA-SARC dataset.

**Fold Number**	**This Paper Model**	**U-Net++**	**DeepLabv3+**	**Attention U-Net**	**TransUNet**
1	0.973	0.952	0.959	0.956	0.954
2	0.971	0.948	0.955	0.951	0.950
3	0.974	0.955	0.961	0.957	0.958
4	0.969	0.946	0.953	0.949	0.947
5	0.975	0.957	0.963	0.960	0.961
6	0.972	0.951	0.958	0.954	0.952
7	0.970	0.949	0.956	0.952	0.950
8	0.976	0.959	0.964	0.962	0.963
9	0.973	0.953	0.959	0.956	0.955
10	0.972	0.950	0.957	0.953	0.951

**Table 3:** Statistical significance test.

**Metrics**	**This Paper Model**	**Attention U-Net**	**p value**
Inference time (ms)	420±15	595±20	<0.001
GPU memory (GB)	3.2	4.3	<0.001
Parameters (M)	28.6	32.5	<0.001

**Table 4:** Diagnosis time.

**Patient Number**	**Manual Diagnosis (min)**	**This Paper Model** **(min)**	**Patient Number**	**Manual Diagnosis (min)**	**This Paper Model** **(min)**
1	15	5	11	16	6
2	17	6	12	19	7
3	16	5	13	14	5
4	18	6	14	20	8
5	14	5	15	17	6
6	19	7	16	15	5
7	16	6	17	18	6
8	17	6	18	16	6
9	15	5	19	17	6
10	18	7	20	19	7

## Data Availability

The OAI-ZIB knee MRI dataset used in this study is available at https://nda.nih.gov/oai/study-details.

## LIST OF ABBREVIATIONS

FPN: Feature Pyramid Network
MRI: Magnetic resonance imaging
